# Supplementary material for: Kv7/KCNQ potassium channels in cortical hyperexcitability and juvenile seizure-related death in Ank2-mutant mice
Source: Nat Commun. 2023 Jun 15;14:3547. doi: 10.1038/s41467-023-39203-z (PMC10272139; doi:10.1038/s41467-023-39203-z)

Supplementary Information

Kv7/KCNQ potassium channels in cortical hyperexcitability and juvenile seizure-related death in Ank2-mutant mice

Hyoseon Oh,[1,2] Suho Lee,[2] Yusang Oh,[1,3] Seongbin Kim,[1] Young Seo Kim,[4] Yeji Yang,[1,5] Woochul Choi,[3] Ye-Eun Yoo,[2] Heejin Cho,[2] Seungjoon Lee,[2] Esther Yang,[6] Wuhyun Koh,[7] Woojin Won,[7] Ryunhee Kim,[2] C. Justin Lee,[7] Hyun Kim,[6] Hyojin Kang,[8] Jin Young Kim,[5] Taeyun Ku,[4] Se-Bum Paik,[3] Eunjoon Kim[1,2,#]

[1]Department of Biological Sciences, Korea Advanced Institute for Science and Technology (KAIST), Daejeon 34141, Korea; [2]Center for Synaptic Brain Dysfunctions, Institute for Basic Science (IBS), Daejeon 34141, Korea; [3]Department of Bio and Brain Engineering, KAIST, Daejeon 34141, Korea; [4]Graduate School of Medical Science and Engineering, KAIST, Daejeon 34141, Korea; [5]Research Center for Bioconvergence Analysis, Korea Basic Science Institute, 162 Yeongudanjiro, Ochang, Cheongju, Chungbuk 28119, Korea; [6]Department of Anatomy and Brain Korea 21 Graduate Program, Biomedical Science, College of Medicine, Korea University, Seoul 02841, Korea; [7]Center for Cognition and Sociality, IBS, Daejeon 34126, Korea; [8]Division of National Supercomputing, Korea Institute of Science and Technology Information, Daejeon 34141, Korea; [#]Corresponding author.

Contents:

Supplementary Figures 1–15

Source Data (uncropped immunoblot images for supplementary figures)

Supplementary figures and legends

Supplementary figure 1

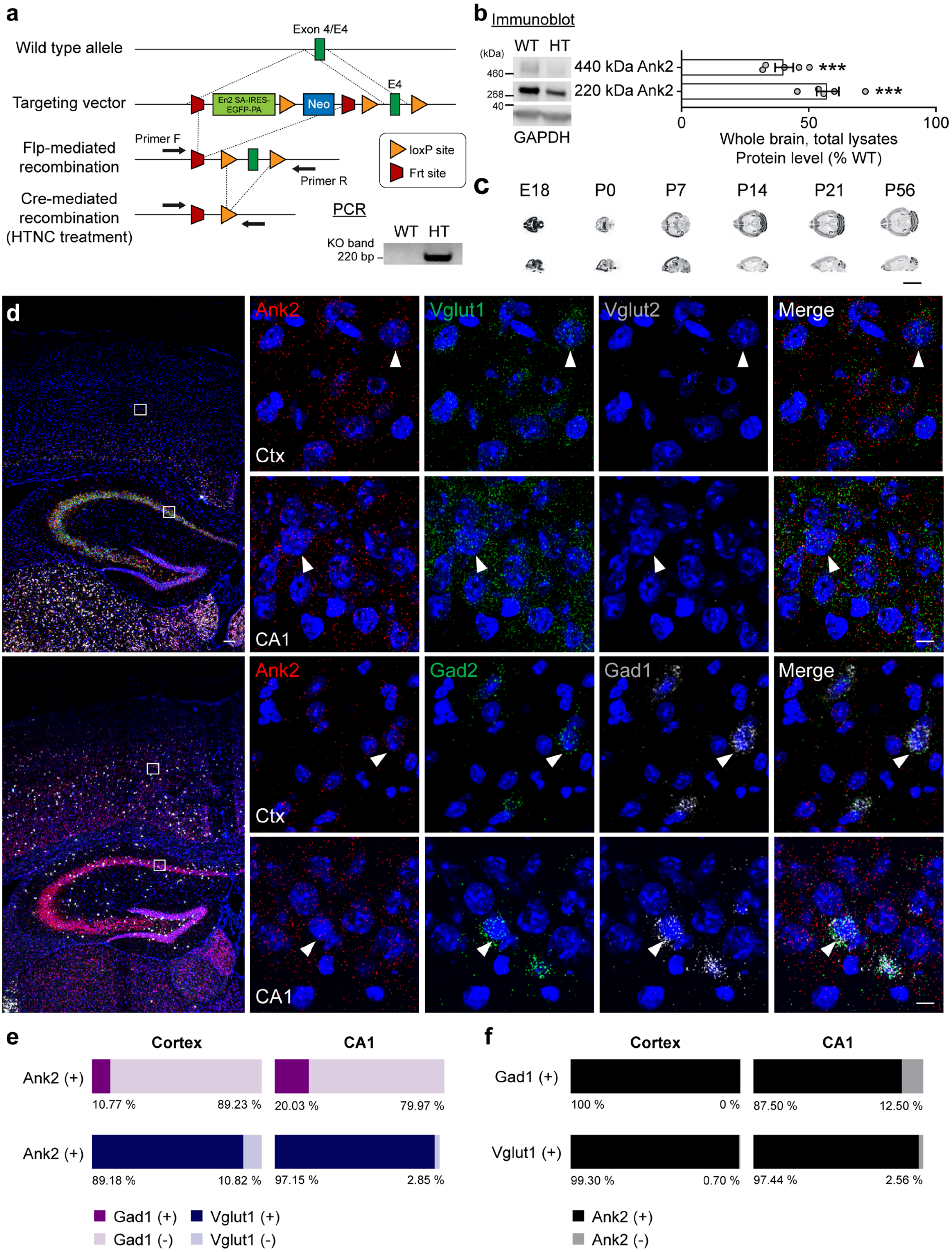

Supplementary Fig. 1. *Ank2*-KO strategy and *Ank2* mRNA distribution.

(a) Schema of *Ank2* conditional knockout (cKO) and global KO strategy targeting exon 4 and their validation by PCR genotyping. *Ank2$^{fl/fl}$* mice derived from Flp-mediated recombination were used to create global Ank2 KO mice under treatment with HTNC (histidine-TAT-NLS-Cre), a His_6-tagged Cre recombinase protein rendered cell-permeable by incorporation of the cell-penetrating TAT peptide. Crossing *Ank2$^{fl/fl}$* mice with *Emx1-Cre* mice (instead of HTNC) was used to generate *Emx1-Cre;Ank2$^{+/fl}$* mice and, subsequently, *Emx1-Cre;Ank2$^{fl/fl}$* mice (Ank2-cKO) mice (not shown here).

(b) Levels of the two Ank2 protein splice variants (220 and 440 kDa) exhibit ~50% decreases in global Ank2 heterozygous KO mice (P56; male; whole brain), as revealed by immunoblot analysis. (n = 5 [WT] and 5 [HT], one sample t-test).

(c) Distribution of *Ank2* mRNA in various brain regions of WT mice, including the cortex, hippocampus, and cerebellum at embryonic day (E) 18, postnatal day (P) 0, P7, P14, P21, and P56, revealed by isotope in situ hybridization. Scale bar, 10 mm.

(d) Detection of *Ank2* mRNAs in Vglut1/2-positive glutamatergic neurons and Gad1/2-positive GABAergic neurons in the neocortical and hippocampal CA1 regions of the mouse brain (P56), as revealed by triple fluorescence in situ hybridization (FISH). Examples of colocalizations at single-cell levels are indicated by arrowheads. DAPI staining of nuclei (blue) was used to indicate individual cell bodies. Scale bar, 100 μm (left) and 10 μm (right).

(e and f) Quantitative analysis of the results in (d). Note that nearly all Vglut1/2-positive glutamatergic neurons and Gad1/2-positive GABAergic neurons are Ank2-positive (f), while Ank2-positive cells are much more frequently Vglut1/2-positive than Gad1/2-positive (e), reflecting the relative abundance of glutamatergic neurons compared to GABAergic neurons in these brain areas. (n = 3 [vGlut1/2 + Ank2] and 3 [Gad1/2 + Ank2]).

Source data for uncropped immunoblot images are provided as a Source Data fil e. The statistical tests involved two-sided analyses. Data are presented as mean v alues +/- SEM. P values in figure panels: *p < 0.05, **p < 0.01, ***p < 0.001, ns, not significant.

Supplementary figure 2

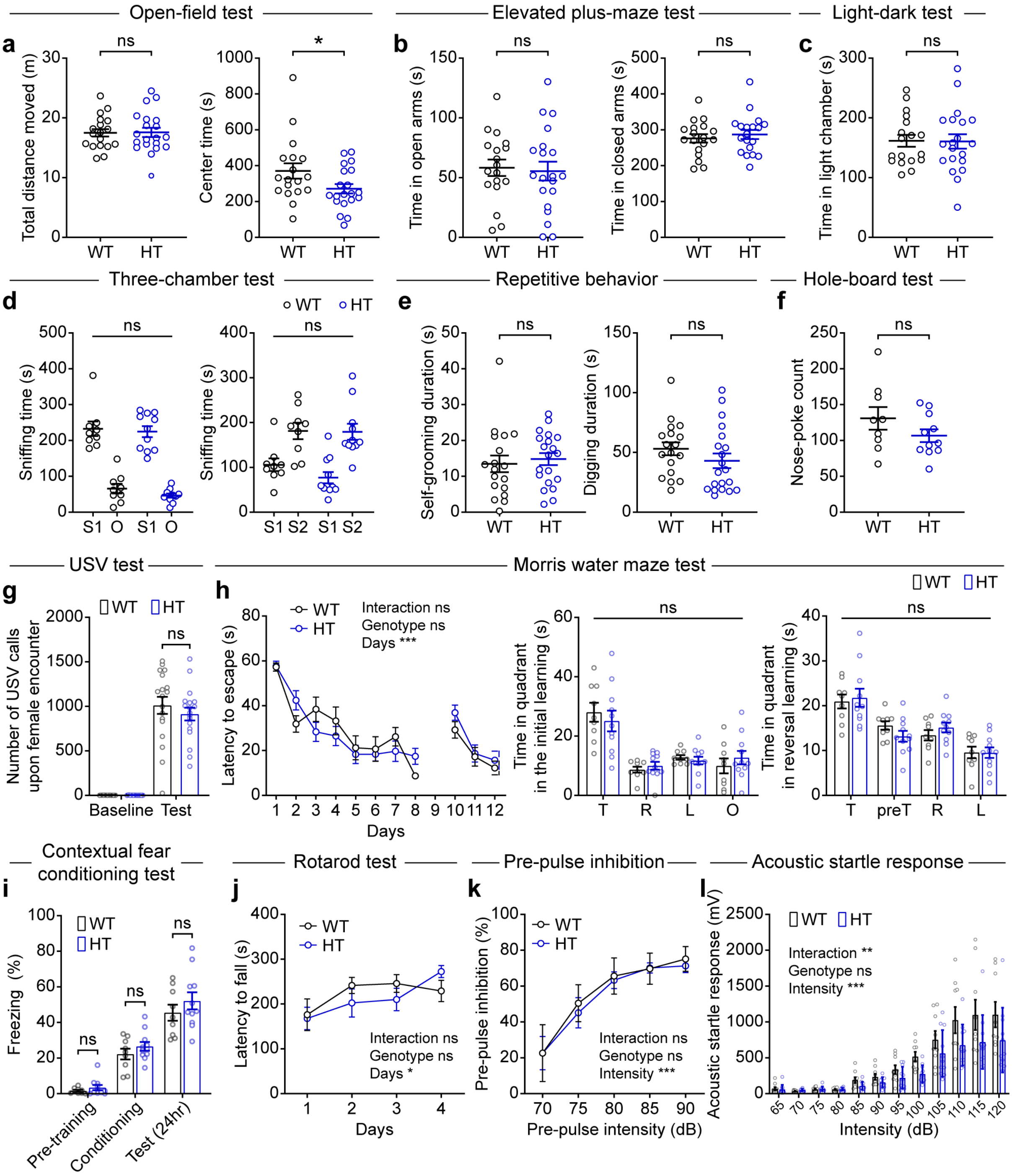

Supplementary Fig. 2. Largely normal behaviors in male *Ank2*$^{+/-}$ mice.

(a) Normal locomotor activity of *Ank2*$^{+/-}$ mice (2–3 months) in the open-field test (100 lux), as shown by distance moved. Note that these mice show moderate anxiety-like behavior, as shown by the slightly decreased center time. (n = 18 mice [WT] and 20

[HT], Student's t-test [total distance moved], Mann-Whitney test [center time]).

(b) Normal anxiety-like behavior in *Ank2*$^{+/-}$ mice (2–3 months) in the elevated plus-maze test (250 lux), as shown by time spent in the open and closed arms. (n = 18 [WT] and 20 [HT], Student's t-test).

(c) Normal anxiety-like behavior in *Ank2*$^{+/-}$ mice (2–3 months) in the light-dark test (600 lux), as shown by time spent in the light box. (n = 18 [WT] and 20 [HT], Student's t-test).

(d) Normal social approach and social-novelty recognition in *Ank2*$^{+/-}$ mice (2–3 months) in the three-chamber test, as shown by time spent in sniffing the targets (O, object; S1/S2, first/second social stranger). (n = 9 [WT] and 11 [HT], two-way ANOVA).

(e) Normal repetitive behaviors in *Ank2*$^{+/-}$ mice (2–3 months) in a new home cage, as shown by self-grooming and digging durations. (n = 18 [WT] and 20 [HT], Mann-Whitney test).

(f) Normal repetitive behavior in *Ank2*$^{+/-}$ mice (2–3 months) in the hole-board test, as shown by nose-poke count. (n = 9 [WT] and 11 [HT], Student's t-test).

(g) Normal social communication in *Ank2*$^{+/-}$ mice (2–3 months) in the courtship behavior test, as shown by number of ultrasonic vocalization (USV) calls emitted by a male encountering a novel female. (n = 9 [WT] and 11 [HT], Mann-Whitney test [base line], Student's t-test [test]).

(h) Normal spatial learning and memory in *Ank2*$^{+/-}$ mice (2–3 months) in the Morris water maze test, as shown by time spent in each quadrant in the first probe (initial learning) and second probe (for reversal learning) tests. T, target quadrant; O, opposite; L, left; R, right; preT, previous target quadrant. (n = 9 [WT] and 11 [HT], Student's t-test).

(i) Normal contextual fear learning and memory in *Ank2*$^{+/-}$ mice (2–3 months), as shown by freezing level. (n = 9 [WT] and 11 [HT], Mann-Whitney test [Pre-training], Student's t-test [Conditioning and Test after 24 hours]).

(j) Normal motor coordination and learning function of *Ank2*$^{+/-}$ mice (2–3 months) as shown by latency to fall in the rotarod test. (n = 7 [WT] and 9 [HT], two-way repeated-measures/RM-ANOVA).

(k) Normal pre-pulse inhibition of *Ank2*$^{+/-}$ mice (2–3 months), as shown by percentage of pre-pulse inhibition plotted against pre-pulse intensity. (n = 8 [WT] and 9 [HT], two-way RM-ANOVA).

(l) Normal acoustic startle response of *Ank2*$^{+/-}$ mice (2–3 months) as shown by startle response plotted against pulse intensity. (n = 9 [WT] and 11 [HT], two-way RM-ANOVA with Sidak's test).

The statistical tests involved two-sided analyses, and adjustments were made for multiple comparisons. Data are presented as mean values +/- SEM. P values in figure panels: *p < 0.05, **p < 0.01, ***p < 0.001, ns, not significant.

Supplementary figure 3

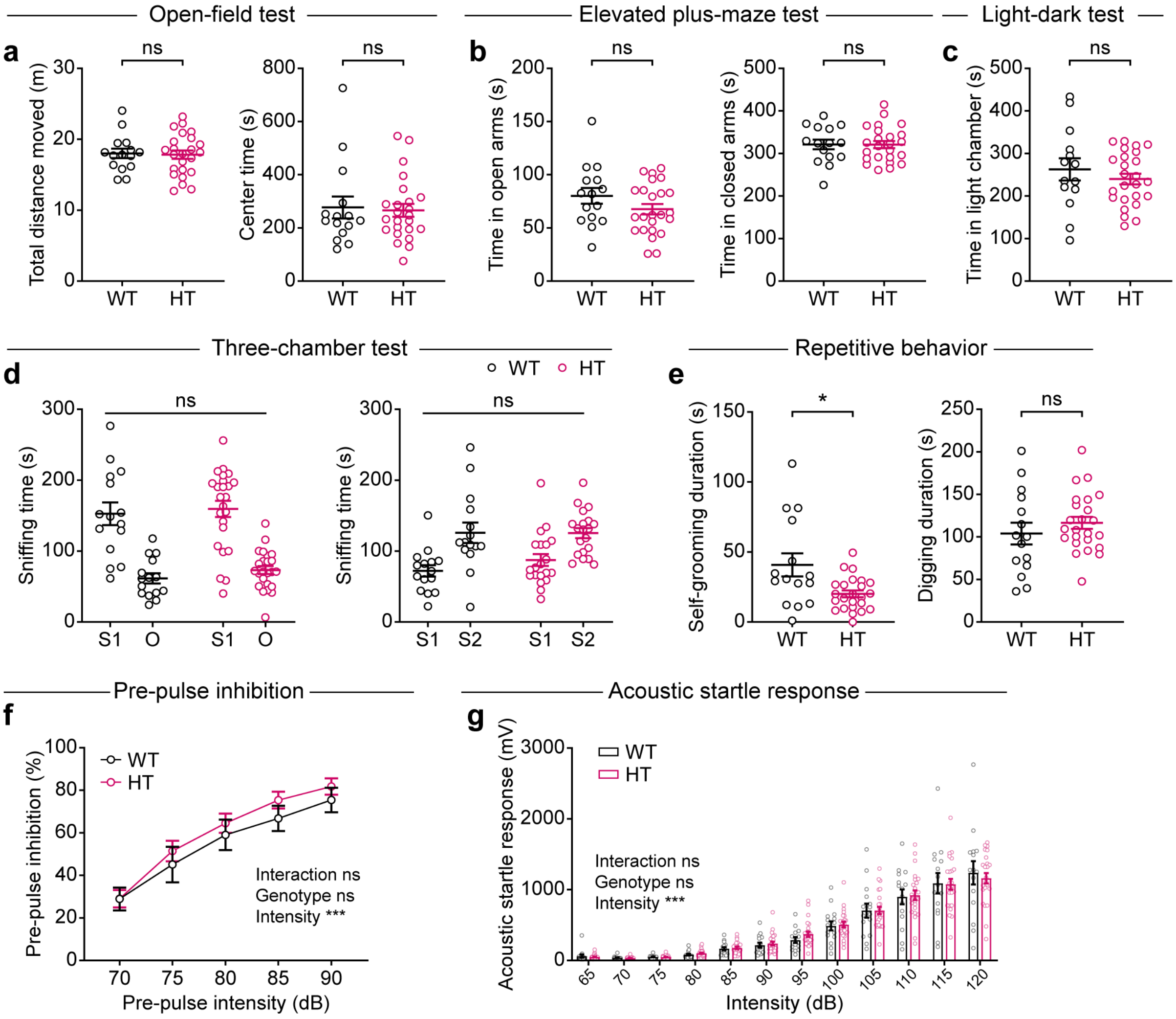

Supplementary Fig. 3. Largely normal behaviors in female *Ank2*$^{+/-}$ mice.

(a) Normal locomotor activity of *Ank2*$^{+/-}$ mice (2–3 months) in the open-field test (100 lux), as shown by distance moved. (n = 15 mice [WT] and 24 [HT], Student's t-test [total distance moved], Mann-Whitney test [center time]).

(b) Normal anxiety-like behavior in *Ank2*$^{+/-}$ mice (2–3 months) in the elevated plus-maze test (250 lux), as shown by time spent in the open and closed arms. (n = 15 [WT] and 24 [HT], Student's t-test).

(c) Normal anxiety-like behavior in *Ank2*$^{+/-}$ mice (2–3 months) in the light-dark test (600 lux), as shown by time spent in the light box. (n = 15 [WT] and 24 [HT], Student's t-test).

(d) Normal social approach and social-novelty recognition in *Ank2*$^{+/-}$ mice (2–3 months) in the three-chamber test, as shown by time spent in sniffing the targets (O,

object; S1/S2, first/second social stranger). (n = 15 [WT] and 24 [HT], two-way ANOVA).

(e) Moderately altered repetitive behaviors in *Ank2*$^{+/–}$ mice (2–3 months) in a new home cage, as shown by decreased self-grooming but normal digging. (n = 15 [WT] and 24 [HT], Welch's t-test [Self-grooming duration], Student's t-test [Digging duration]).

(f) Normal pre-pulse inhibition of *Ank2*$^{+/–}$ mice (2–3 months), as shown by percentage of pre-pulse inhibition plotted against pre-pulse intensity. (n = 15 [WT] and 24 [HT], two-way RM-ANOVA).

(g) Normal acoustic startle response of *Ank2*$^{+/–}$ mice (2–3 months) as shown by startle response plotted against pulse intensity. (n = 15 [WT] and 24 [HT], two-way RM-ANOVA).

The statistical tests involved two-sided analyses, and adjustments were made for multiple comparisons. Data are presented as mean values +/- SEM. P values in figure panels: *p < 0.05, **p < 0.01, ***p < 0.001, ns, not significant.

Supplementary figure 4

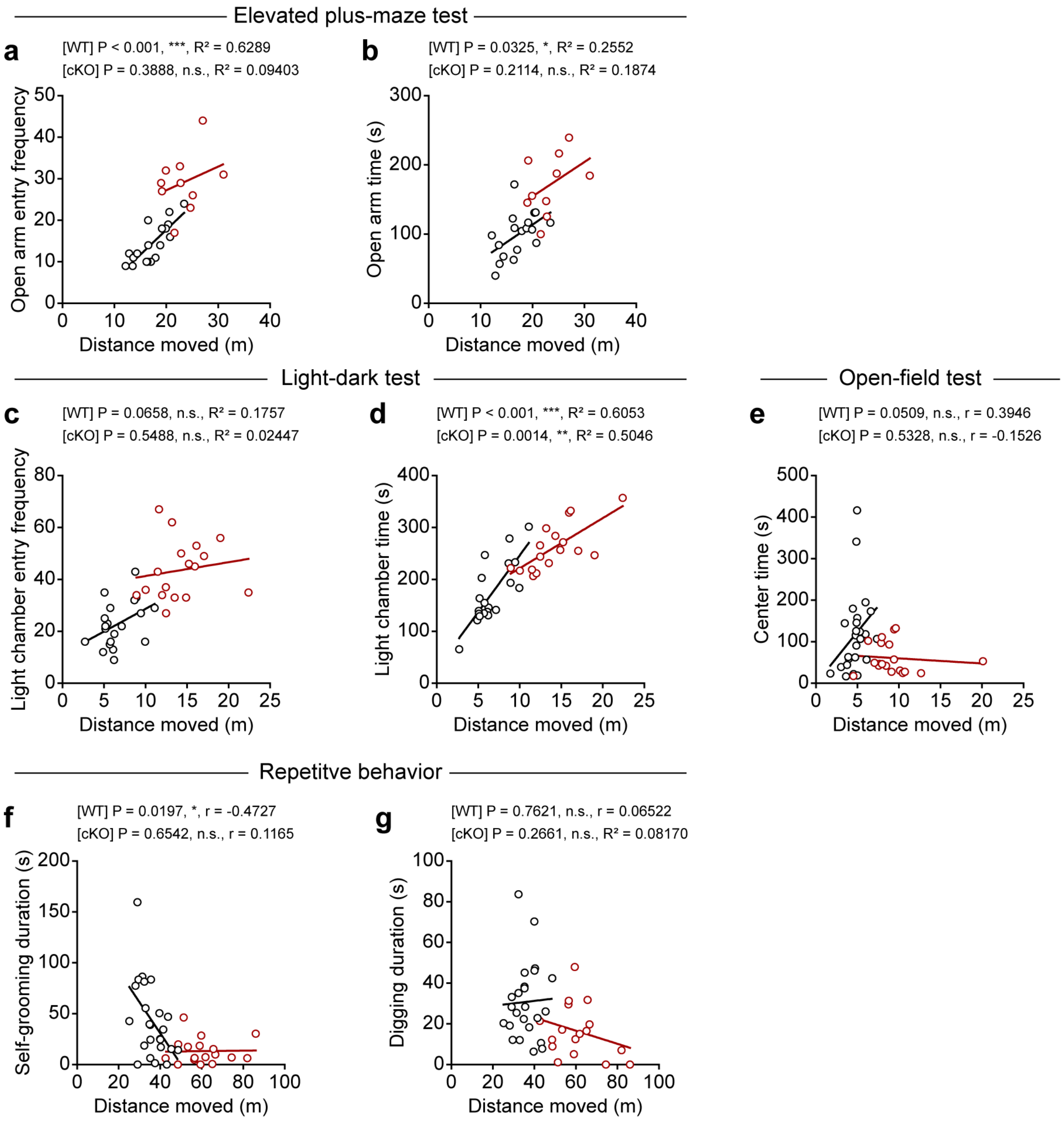

Supplementary Fig. 4. Correlation between hyperactivity and anxiety-like behaviors in Ank2-cKO mice.

(a and b) Correlation between locomotor activity (distance moved) and anxiolytic-like behaviors (open-arm entry/time) of WT and Ank2-cKO mice (P24–25) in the elevated plus-maze test. (n = 18 [WT], 10 [cKO], Pearson test).

(c and d) Correlation between locomotor activity and anxiolytic-like behaviors (light-chamber entry/time) of WT and Ank2-cKO mice (P19–21) in the light-dark test. (n = 20 [WT], 17 [cKO], Pearson test).

(e) Correlation between locomotor activity and anxiety-like behaviors (center time) of WT and Ank2-cKO mice (P19–21) in the open-field test. (n = 25 [WT], 19 [cKO], Spearman test).

(f and g) Correlation between locomotor activity and repetitive behaviors (self-grooming and digging) of WT and Ank2-cKO mice (P25–27) in the open-field test. (n = n = 24 [WT], 17 [cKO], Spearman test [self-grooming_WT/cKO and digging duration_WT], Pearson test [digging duration_cKO]).

The statistical tests involved two-sided analyses.

Supplemenatry figure 5

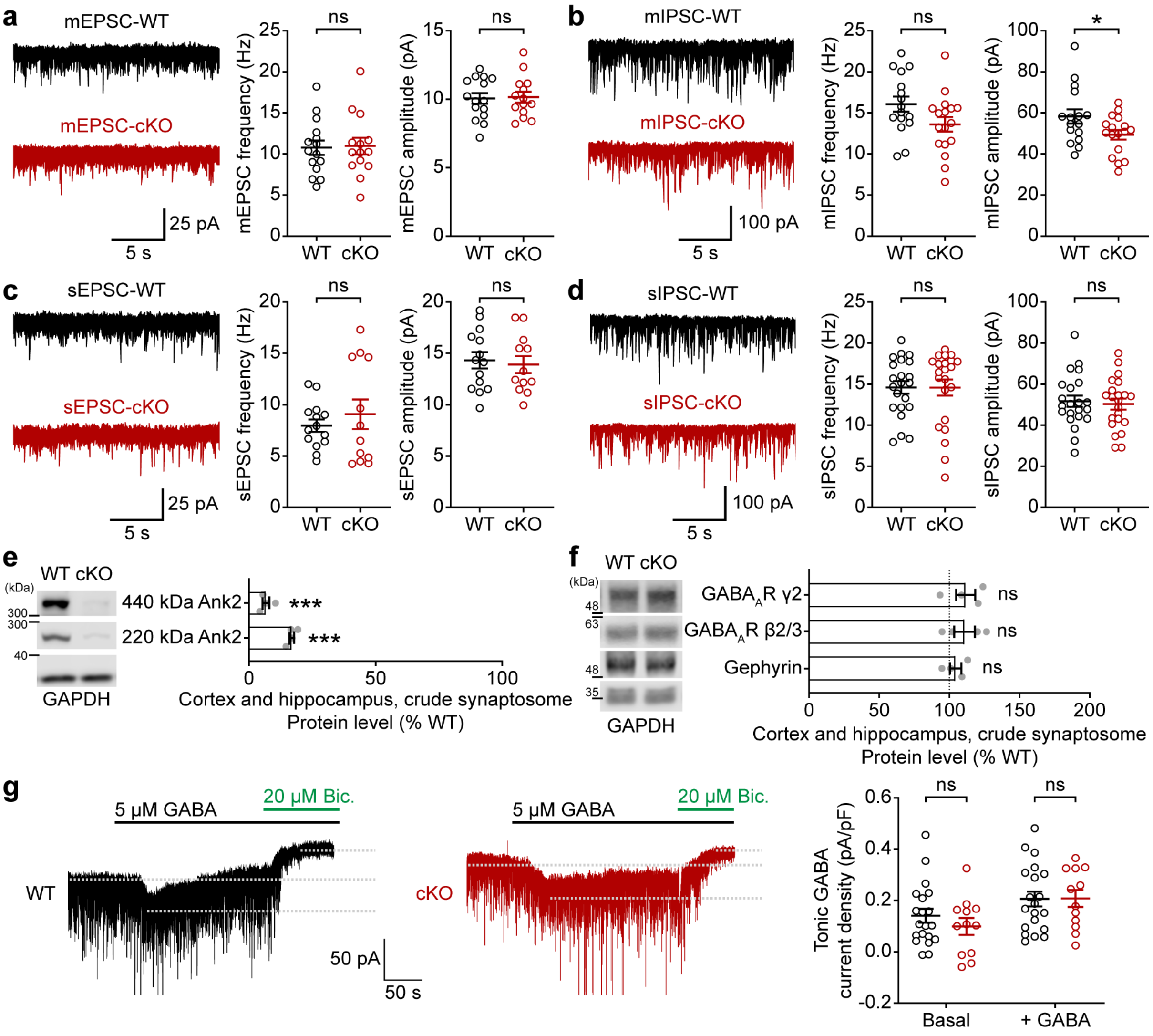

Supplementary Fig. 5. Largely normal spontaneous synaptic transmissions and tonic GABA currents in the Ank2-cKO SSC.

(a) Normal frequency and amplitude of mEPSCs in Ank2-cKO SSC layer 2/3 pyramidal neurons (P19–23). (n = 15 neurons from 7 mice [WT], 14, 5 [cKO], Student's t-test).

(b) Moderately decreased amplitude but not frequency of mIPSCs in Ank2-cKO SSC layer 2/3 pyramidal neurons (P19–23). (n = 16, 3 [WT], 17, 3 [cKO], Student's t-test).

(c) Normal frequency and amplitude of sEPSCs in Ank2-cKO SSC layer 2/3 pyramidal neurons (P19–23). (n = 14, 4 [WT], 12, 4 [cKO], Mann-Whitney test [Frequency], Student's t-test [Amplitude]).

(d) Normal frequency and amplitude of sIPSCs in Ank2-cKO SSC layer 2/3 pyramidal

neurons (P19–23). (n = 22, 4 [WT], 22, 4 [cKO], Mann-Whitney test [Frequency], Student's t-test [Amplitude]).

(e and f) Normal levels of $GABA_A$ receptor subunits β2/3 and γ3 in Ank2-cKO cortex and hippocampus (P19–22). Note that the levels of the 440 and 220 kDa Ank2 protein variants in Ank2-cKO mice were decreased to ~10% and ~20%, respectively, of the WT levels. The residual proteins likely represent those expressed in non-excitatory neurons, such as GABA neurons. Immunoblot signals obtained for Ank2 or $GABA_A$ receptor subunits were normalized to GAPDH signals. (n = 4 mice [WT], 4 [cKO], One sample t-test).

(g) Normal tonic GABA currents under baseline and GABA-treated conditions in Ank2-cKO SSC layer 2/3 pyramidal neurons (P19–23). (n = 20, 3 [WT], 12, 3 [cKO], Student's t-test).

Source data for uncropped immunoblot images are provided as a Source Data fil e. The statistical tests involved two-sided analyses. Data are presented as mean v alues +/- SEM. P values in figure panels: *p < 0.05, **p < 0.01, ***p < 0.001, ns, not significant.

Supplemenatry figure 6

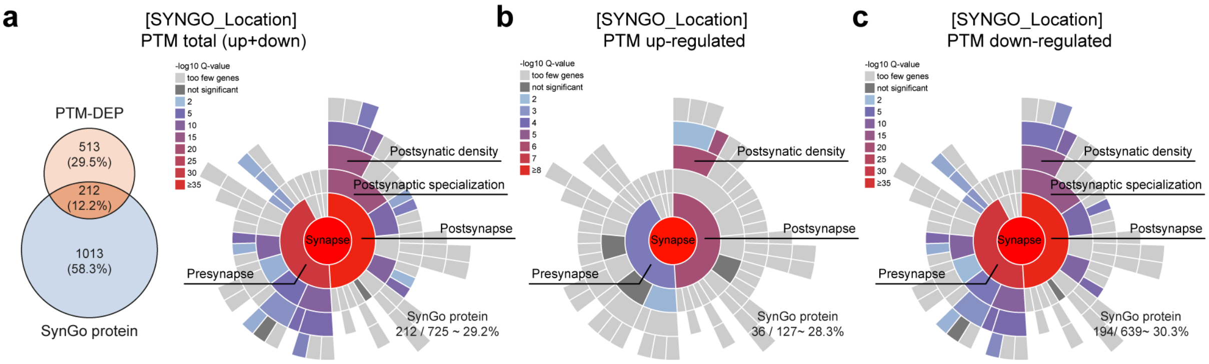

Supplementary Fig. 6. SynGO analyses of the PTM proteins from Ank2-cKO mice.

(a–c) SynGO analyses of the PTM proteins from Ank2-cKO mice. Note that both up- and down-PTM proteins are more strongly enriched for postsynaptic SynGO proteins relative to presynaptic SynGO proteins.

Supplementary figure 7

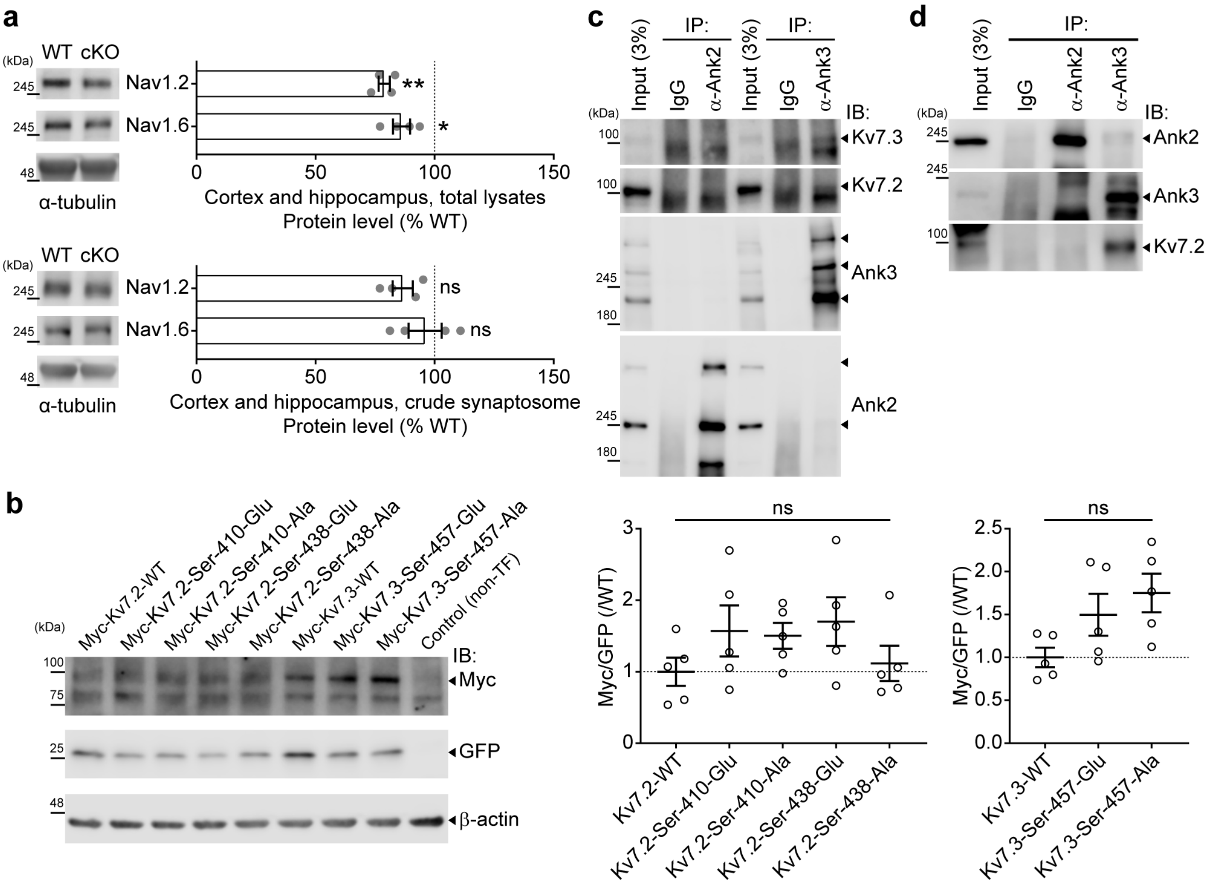

Supplementary Fig. 7. Tests of Ank2/3-Kv7 interactions, effects of Kv7 phosphorylation, and effects of Ank2 cKO on Nav channel levels.

(a) Normal synaptosomal but moderately decreased total levels of Nav1.2 and Nav1.6 sodium channel subunits in the Ank2-cKO cortex and hippocampus (P19–23). Nav1.2/6 signals were normalized to those of α-tubulin. (n = 4 mice [WT, cKO], one-sample t-test).

(b) Effects of Kv7.2/3 point mutations (phospho-mimic and non-phosphorylatable; Kv7.2-Ser-410/438-Glu/Ala and Kv7.3-Ser-457-Glu/Ala) on protein stability in cultured mouse cortical neurons (DIV 5–14). Kv7.2/3 (WT or mutants) were co-expressed with GFP in cultured mouse cortical neurons at DIV 5 and compared total protein levels at DIV 14–17. GFP was co-expressed with Kv7.2/3 to normalize exogenous protein expressions. (n = 5 independent experiments, one-way ANOVA).

(c and d) Undetectable biochemical association between Ank2 and Kv7.2/3 or between Ank2 and Ank3 in cultured hippocampal neurons (DIV 14) (c) and the WT mouse brain (P21; d). IP, immunoprecipitation; IB, immunoblot. (n = 4 mice [WT, cKO]).

Source data for uncropped immunoblot images are provided as a Source Data file. The statistical tests involved two-sided analyses. Data are presented as mean values +/- SEM. P values in figure panels: *p < 0.05, **p < 0.01, ***p < 0.001, ns, not significant.

Supplementary figure 8

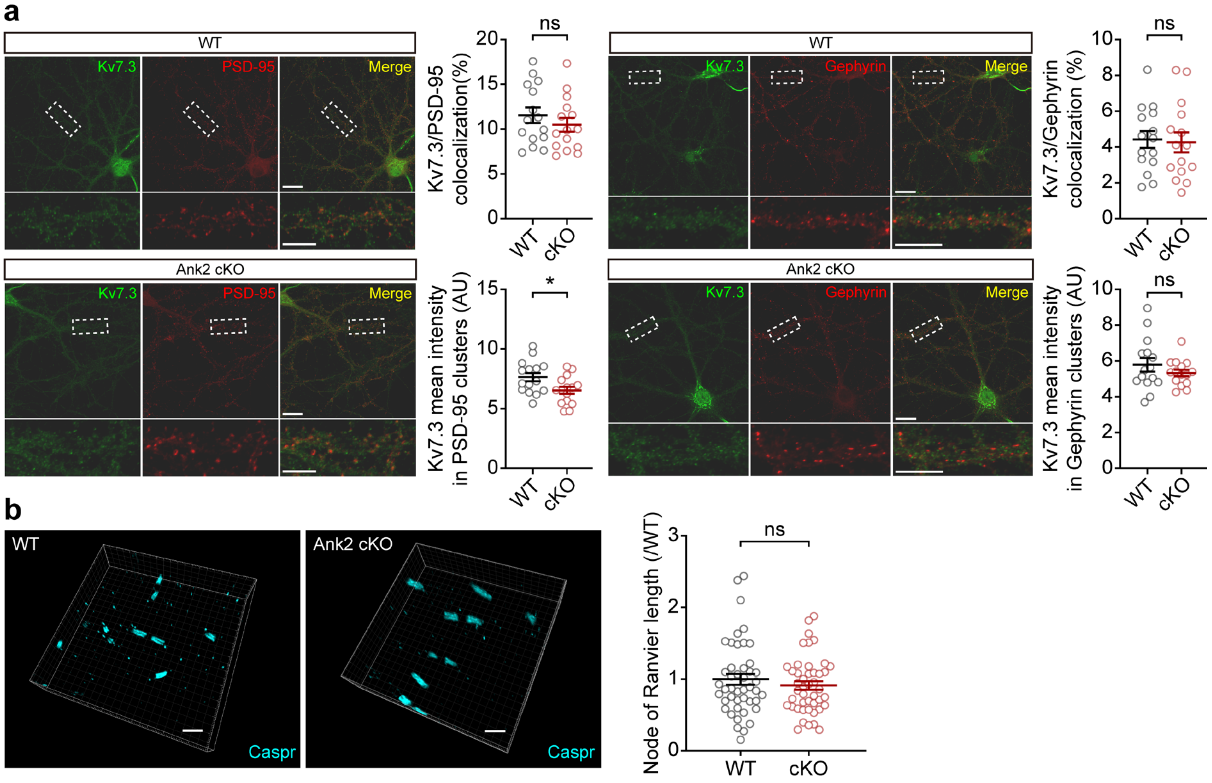

Supplementary Fig. 8. Effects of Ank2 cKO on synaptic Kv7 localization and the length of nodes of Ranvier.

(a) Effects of Ank2 cKO on the localization of Kv7.3 at excitatory and inhibitory synapses in cultured cortical neurons (DIV 17). Note that the colocalization, determined by colocalized areas, between Kv7.3 and PSD-95 (an excitatory synapse marker) is not changed, whereas the mean intensity of Kv7.3 at the colocalized area is moderately decreased. (n = 15 images from 3 independent experiments [WT and cKO], Student's t-test except for [Kv7.3 mean intensity in Gephyrin clusters] for Welch's t-test). Scale bar, 20 μm (up), 10 μm (down).

(b) Effects of Ank2 cKO on the length of nodes of Ranvier in expanded mouse brain slices (P21; see below for details on brain expansion) visualized by immunostaining for Caspr (a marker for paranodes flanking the node of Ranvier). (n = 45 images from 3 mice [WT and cKO], Mann-Whitney test). Scale bar, 5 μm.

The statistical tests involved two-sided analyses. Data are presented as mean valu es +/- SEM. P values in figure panels: *p < 0.05, **p < 0.01, ***p < 0.001, ns, not significant.

Supplementary figure 9

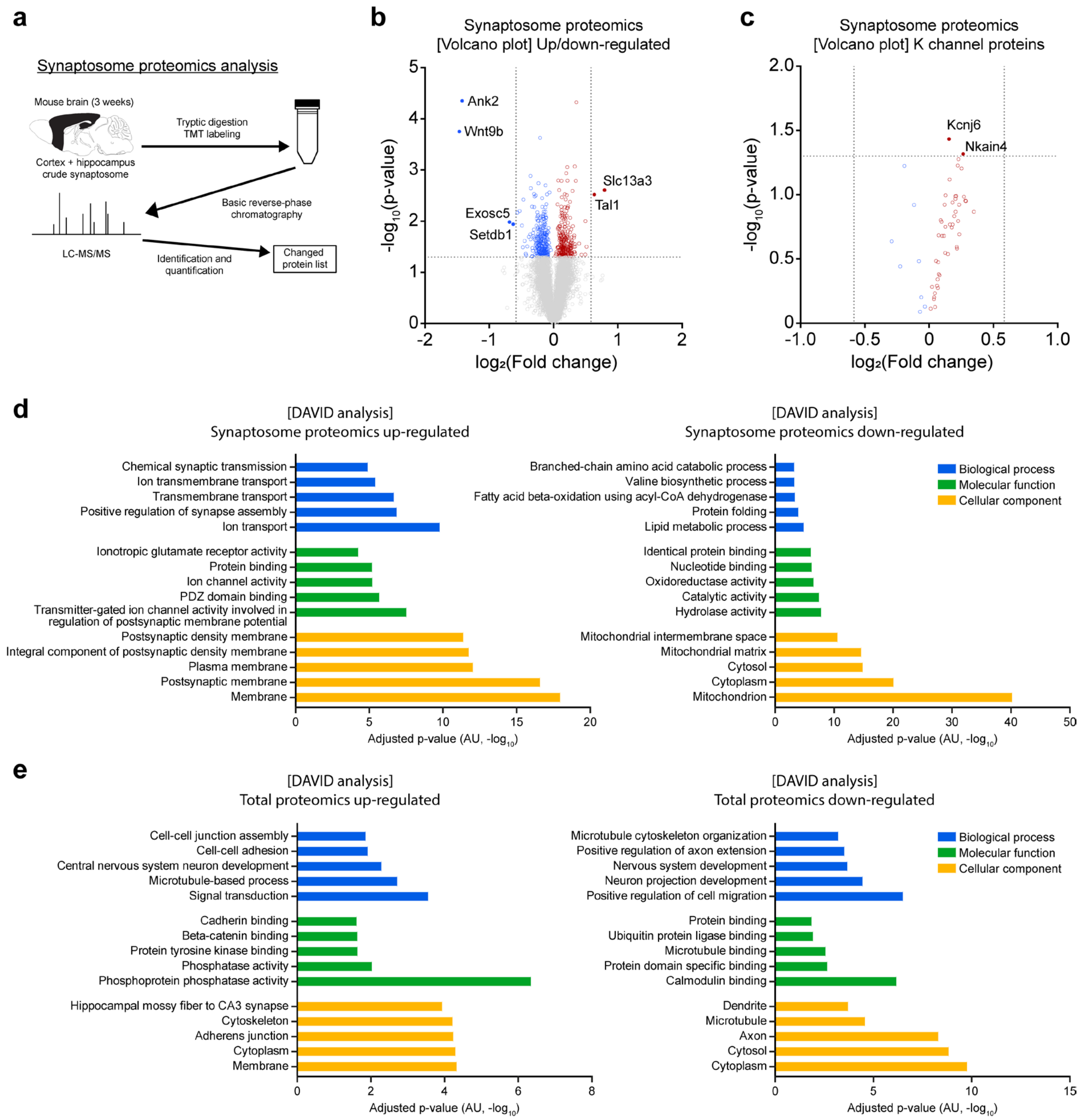

Supplementary Fig. 9. Proteomic analyses of synaptosomal proteins in the Ank2-cKO brain (volcano plot and DAVID).

(a) Diagram depicting procedures of proteomic analysis of synaptosomal (crude synaptosome; P2 fraction) proteins in Ank2-cKO mice (cortex + hippocampus; P19–22).

(b and c) Volcano plots highlighting the synaptosomal (crude synaptosome; P2 fraction) proteins with significant fold changes (>1.5) and p values (< 0.05, Welch's t-test) in Ank2-cKO mice (cortex + hippocampus; P19–22) and the presence of a small

number of potassium channel-related proteins but not Kv7.2/3 proteins in the synaptosomal DEPs ($p < 0.05$ [Welch's t-test] but not fold change > 1.5). ($n = 3$ mice [WT, cKO]).

(d and e) Gene ontology terms derived from DAVID analyses of synaptosomal and total DEPs from Ank2-cKO mice ($p < 0.05$, Welch's t-test).

Supplementary figure 10

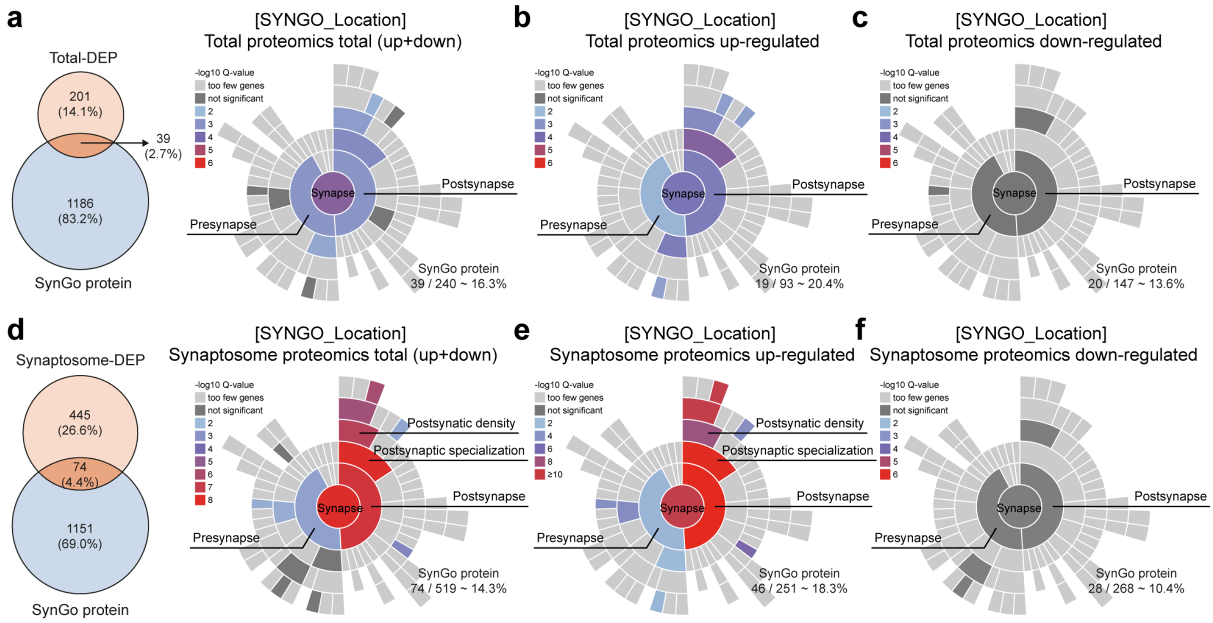

Supplementary Fig. 10. SynGO analyses of total and synaptosomal proteins in the Ank2-cKO brain.

(a–f) SynGO analysis of total and synaptosomal DEPs ($p < 0.05$, Welch's t-test) from the Ank2-cKO brain (cortex + hippocampus; P19–22).

Supplementary figure 11

a [PSEA analysis] C5 - Total proteomics

Total	Down-regulated			Up-regulated		
	Term	FDR	NES	Term	FDR	NES
GO BP	mRNA metabolic process	0	-3.59	Cation transmembrane transport	0	5.74
	RNA processing	0	-3.52	Cellular respiration	0	5.14
	Regulation of mRNA metabolic process	0	-3.51	Inorganic ion transmembrane transport	0	4.86
	Cytoplasmic translation	0	-3.41	ATP sysnthesis coupled electron transport	0	4.66
	Regulation of RNA splicing	0	-3.35	Electron transport chain	0	4.56
GO CC	Ribonucleoprotein complex	0	-3.82	Organelle inner membrane	0	5.74
	Cytoplasmic stress granule	0	-3.32	Mitochondrial protein containing complex	0	5.14
	Microtubule	0	-3.18	Inner mitochondrial membrane protein complex	0	4.86
	Ribonucleoprotein granule	0	-3.17	Respirasome	0	4.66
	Cytosolic ribosome	0	-3.10	Transporter complex	0	4.56
GO MF	mRNA binding	0	-3.18	Active transmembrane transporter activity	0	6.16
	mRNA 3'UTR binding	0.0081	-2.62	Cation transmembrane transporter activity	0	5.23
	Structural constituent of ribosome	0.0060	-2.61	Ion transmembrane transporter activity	0	5.03
	Extracellular matrix structural constituent	0.0067	-2.57	Primary active transmembrane transporter activity	0	4.87
	Chromatin binding	0.0131	-2.44	Inorganic molecular entity transmembrane transporter activity	0	4.75

b [PSEA analysis] C5 - Synaptosome proteomics

Total	Down-regulated			Up-regulated		
	Term	FDR	NES	Term	FDR	NES
GO BP	Organic acid metabolic process	0	-5.44	Synaptic signaling	0	6.27
	Small molecule catabolic process	0	-4.91	Cation transmembrane transport	0	5.59
	Cellular amino acid metabolic process	0	-4.87	Cell-cell adhesion via plasma membrane adhesion molecules	0	5.51
	Alpha amino acid metabolic process	0	-4.39	Cell-cell adhesion	0	5.50
	Macroautophagy	0	-4.17	Inorganic ion transmembrane transport	0	5.42
GO CC	Mitochondrial matrix	0	-5.66	Synaptic membrane	0	8.14
	Ficolin 1 rich granule lumen	0	-4.67	Postsynaptic membrane	0	7.68
	Microtubule	0	-4.55	Intrinsic component of synaptic membrane	0	7.35
	Proteasome complex	0	-4.25	Intrinsic component of postsynaptic membrane	0	7.08
	Ficolin 1 rich granule	0	-4.19	Receptor complex	0	6.92
GO MF	Oxidoreductase activity	0	-3.86	Molecular transducer activity	0	8.39
	GTPase binding	0	-3.36	Ion transmembrane transporter activity	0	7.57
	Hydrolase activity acting on acid anhydrides	0	-3.33	Inorganic molecular entity transmembrane transporter activity	0	6.80
	NAD binding	0	-3.33	Cation transmembrane transporter activity	0	6.77
	Magnesium ion binding	0	-3.16	Metal ion transmembrane transporter activity	0	6.42

c [PSEA analysis] Enrichment map

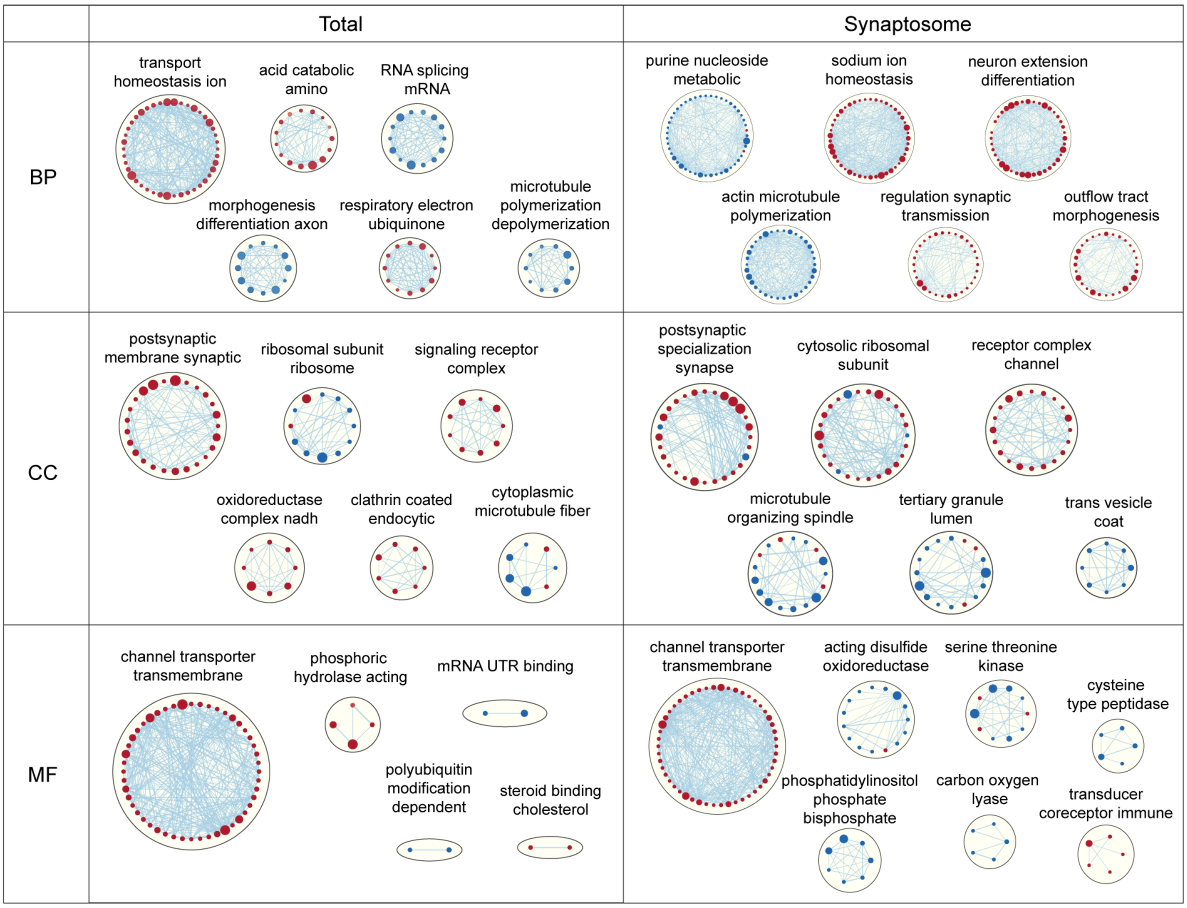

Supplementary Fig. 11. PSEA of total and synaptosomal proteins in the Ank2-cKO brain (gene ontology).

(a–c) Gene set enrichment analysis (GSEA) of total and synaptosomal DEPs from the Ank2-cKO brain (termed PSEA) for the gene sets in the gene ontology domain (C5), as shown by top-five enriched gene sets and the clusters of enriched gene sets produced using the Cytoscape EnrichmentMap App. BP, biological process; CC, cellular component; MF, molecular function. (n = 3 mice [WT, cKO]).

Supplementary figure 12

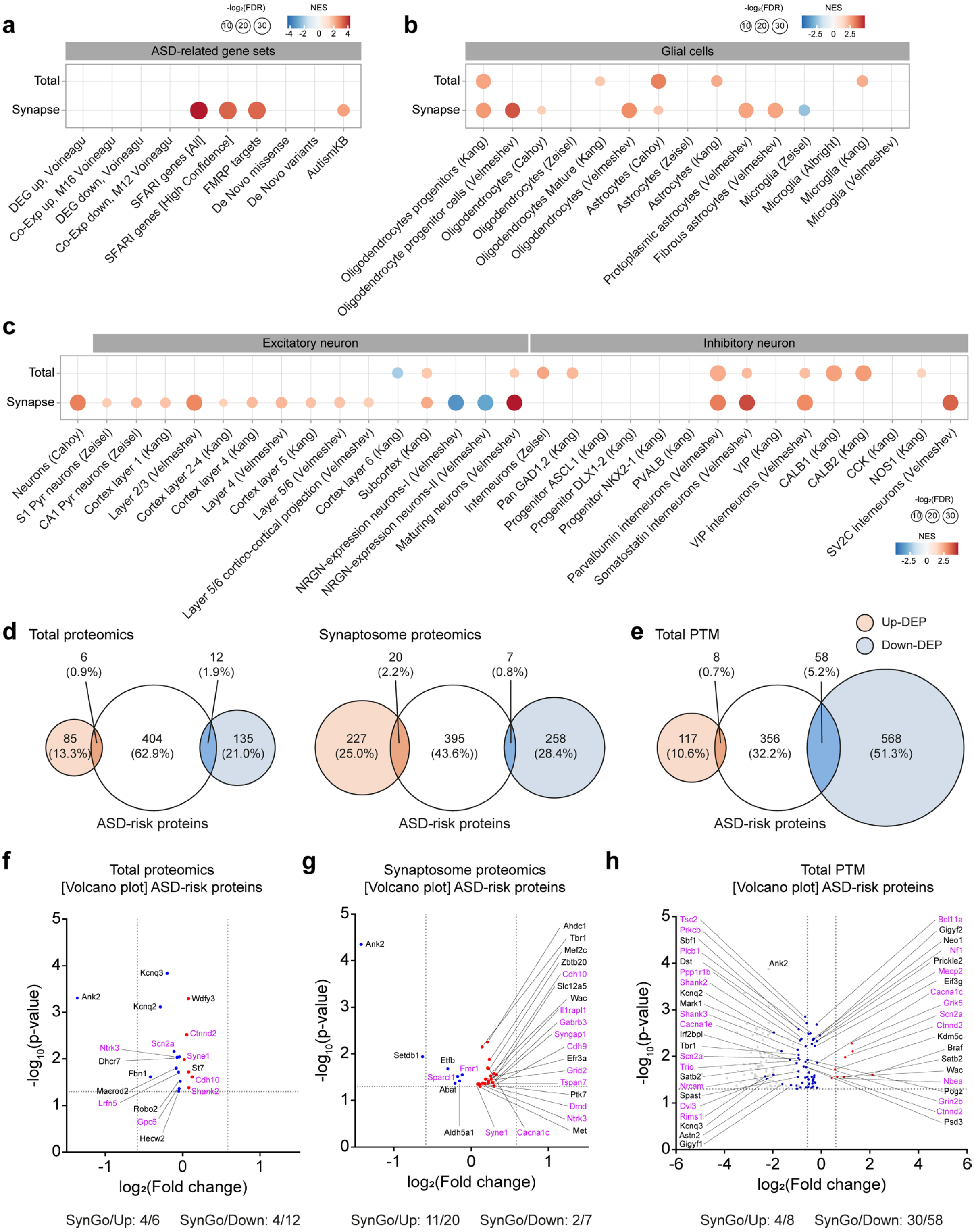

Supplementary Fig. 12. PSEA of total and synaptosomal proteins in the Ank2-cKO brain (ASD-related).

(a) PSEA of total and synaptosomal DEPs from the Ank2-cKO brain for ASD-related sets (DEG Up, Co-Exp Up M16, DEG Down, and Co-Exp Down M12) and ASD-risk gene sets (SFARI [all, high confidence], FMRP Targets, De Novo Missense, De Novo Variants, AutismKB). (n = 3 mice [WT, cKO]).

(b and c) PSEA of total and synaptosomal DEPs from the Ank2-cKO brain for single-cell-specific gene sets known to be upregulated and downregulated in ASD (neurons/oligodendrocytes and astrocytes/microglia, respectively). (n = 3 mice [WT, cKO]). (n = 3 mice [WT, cKO]).

(d and e) Venn diagrams showing overlaps between total/synaptosomal/PTM DEPs (up and down) and ASD-risk proteins (SFARI).

(f–h) Volcano plots highlighting total/synaptosomal/PTM DEPs ($p < 0.05$, Welch's t-test) overlapping with ASD-risk proteins (SFARI). Upregulated, downregulated, and SynGO proteins are indicated in red, blue, and purple, respectively.

Supplementary figure 13

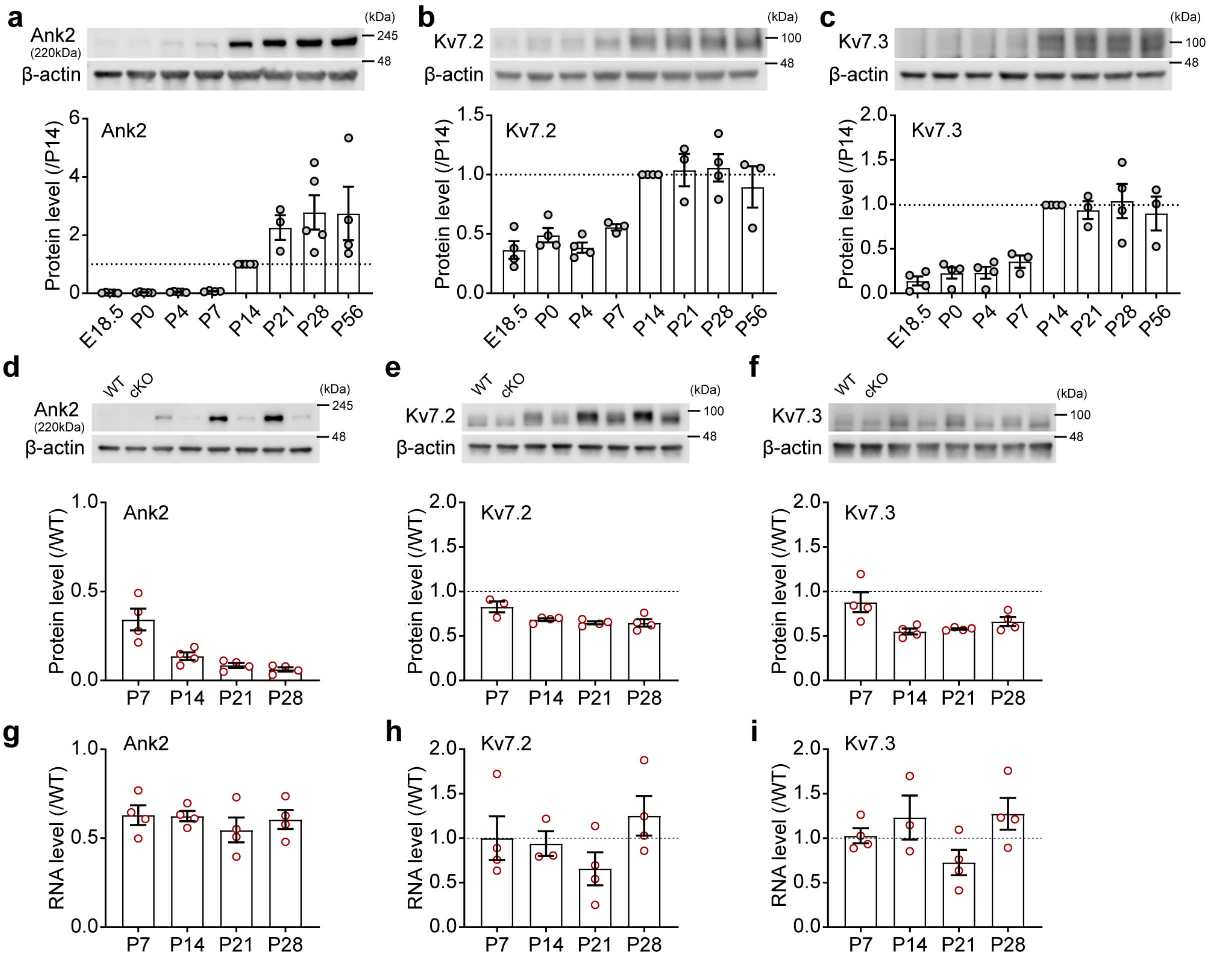

Supplementary Fig. 13. Age-dependent changes in Ank2 and Kv7.2/3 protein levels in WT and Ank2-cKO mice.

(a–c) Expression levels of Ank2 and Kv7.2/3 proteins during the indicated pre- and postnatal stages in WT mice, as revealed by immunoblot analyses (whole brain; Ank2 [n = 5 mice except for 4 for P7/56 and 3 for P21], Kv7.2 and Kv7.3 [n = 4 except for 3 for P7/21/56]).

(d–f) Expression levels of Ank2 and Kv7.2/3 proteins during the indicated postnatal stages in Ank2-cKO mice, as revealed by immunoblot analyses (cortex; Ank2 and Kv7.3 [n = 4 mice], Kv7.2 [n = 4 except for 3 for P7]).

(g–i) Expression levels of Ank2 and Kv7.2/3 mRNAs during the indicated postnatal stages in Ank2-cKO mice, as revealed by RT-qPCR analyses (cortex; Ank2 [n = 4 mice], Kv7.2 and Kv7.3 [n = 4 except for 3 for P14]).

Source data for uncropped immunoblot images are provided as a Source Data file. Data are presented as mean values +/- SEM.

Supplementary figure 14

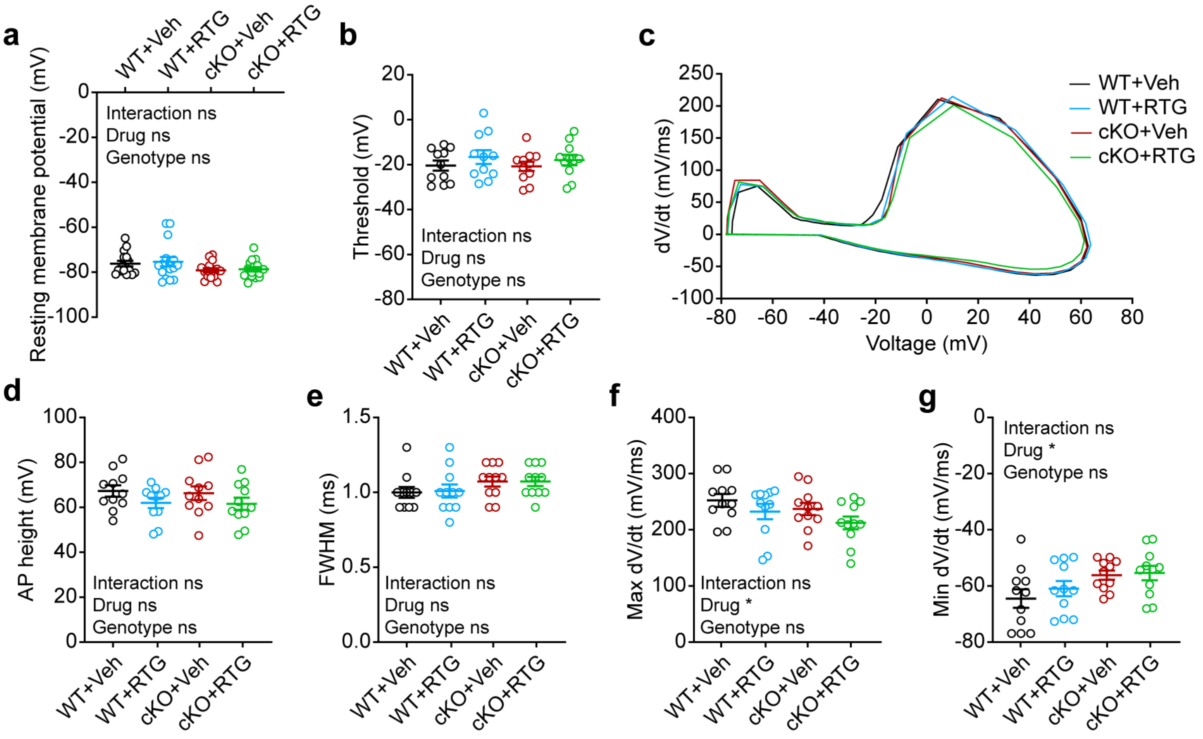

Supplementary Fig. 14. Unaffected AP shapes in retigabine-treated Ank2-cKO mice.

(a) Retigabine treatment (starting from P16–17) does not affect resting membrane potential in Ank2-cKO or WT SSC layer 2/3 pyramidal neurons. (n = 16 neurons/10 mice [WT- Veh/vehicle], 16,10 [WT-RTG/retigabine], 16,9 [cKO-Veh], 16,9 [cKO-RTG], two-way RM-ANOVA).

(b–g) Retigabine treatment (starting from P16–17) does not affect AP threshold or AP shape-related parameters (height, width, and time-dependent voltage changes of APs [dV/dt; maximal/depolarizing and minimal/repolarizing]) in Ank2-cKO or WT SSC layer 2/3 pyramidal neurons. (n = 11,6 [WT-Veh], 11,6 [WT-RTG], 11,7 [cKO-Veh], 11,7 [cKO-RTG], two-way RM-ANOVA).

The statistical tests involved two-sided analyses, and adjustments were made for multiple comparisons. Data are presented as mean values +/- SEM. P values in figure panels: *p < 0.05, **p < 0.01, ***p < 0.001, ns, not significant.

Supplementary figure 15

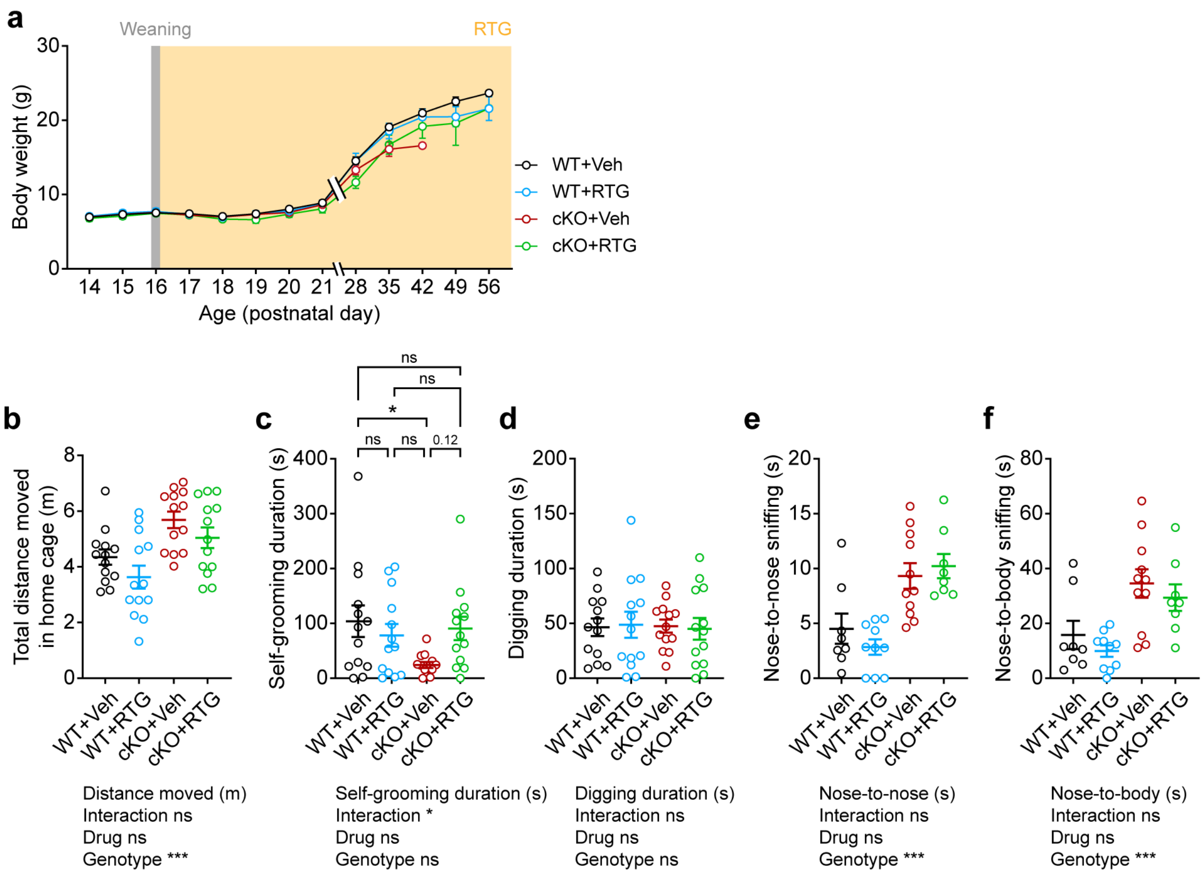

Supplementary Fig. 15. Chronic retigabine treatment does not affect body weights, locomotion, repetitive behavior, or social interaction in Ank2-cKO mice.

(a) Chronic retigabine treatment (from P16–17) does not affect body weights in Ank2-cKO or WT mice (P20–22). (n = 11 mice [WT-Veh/vehicle], 12 [WT-RTG/retigabine], 11 [cKO-Veh], 16 [cKO-RTG]).

(b) Acute retigabine treatment (5 mg/kg) does not affect the locomotor activity of Ank2-cKO or WT mice (P25-26) in home cages. (n = 13 [WT-Veh], 13 [WT-RTG], 13 [cKO-Veh], 13 [cKO-RTG], two-way ANOVA).

(c and d) Acute retigabine treatment (5 mg/kg) does not affect repetitive behaviors (self-grooming and digging) of Ank2-cKO or WT mice (P25-26) in home cages. (n = 13 [WT-Veh], 13 [WT-RTG], 13 [cKO-Veh], 13 [cKO-RTG], two-way ANOVA [Digging duration], with Tukey's test [Self-grooming duration]).

(e and f) Acute retigabine treatment (5 mg/kg) does not affect juvenile play (nose-to-nose and nose-to-body interactions) in Ank2-cKO or WT mice (P23–24). (n = 8 [WT-Veh], 10 [WT-RTG], 11 [cKO-Veh], 8 [cKO-RTG], two-way ANOVA).

The statistical tests involved two-sided analyses, and adjustments were made for multiple comparisons. Data are presented as mean values +/- SEM. P values in figure panels: *p < 0.05, **p < 0.01, ***p < 0.001, ns, not significant.

Source data (uncropped immunoblot images for supplementary figures)

Fig. S1 b uncropped images

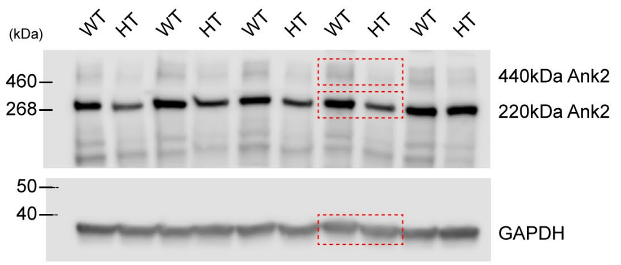

Fig. S5 e uncropped images

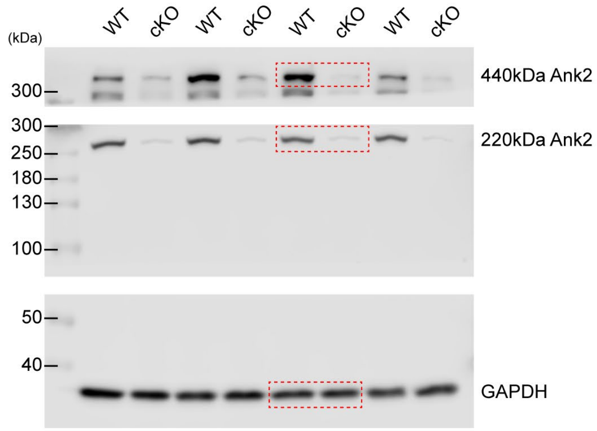

Fig. S5 f uncropped images

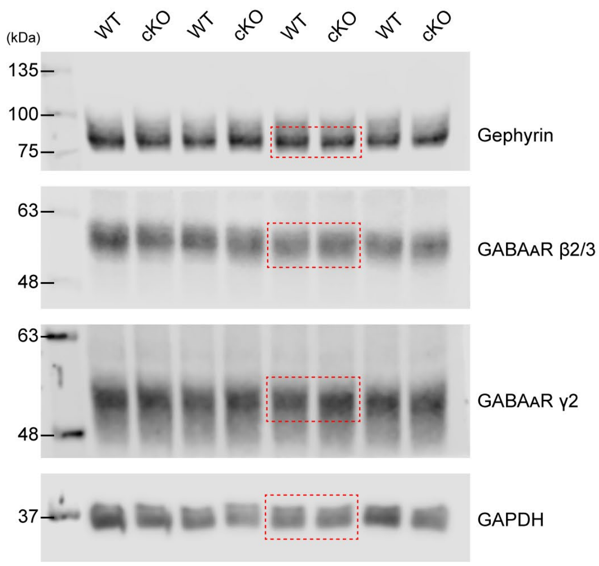

Fig. S7 a uncropped images

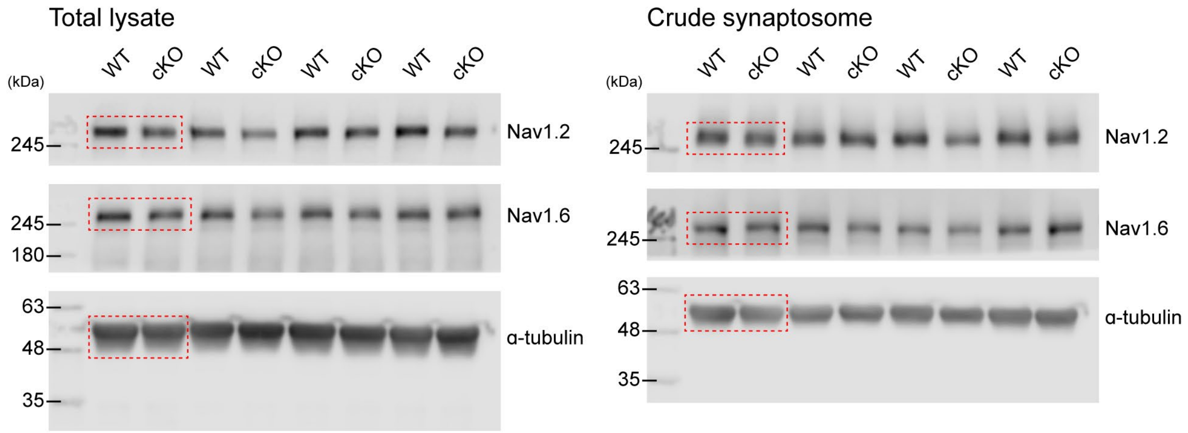

Fig. S7 b uncropped images

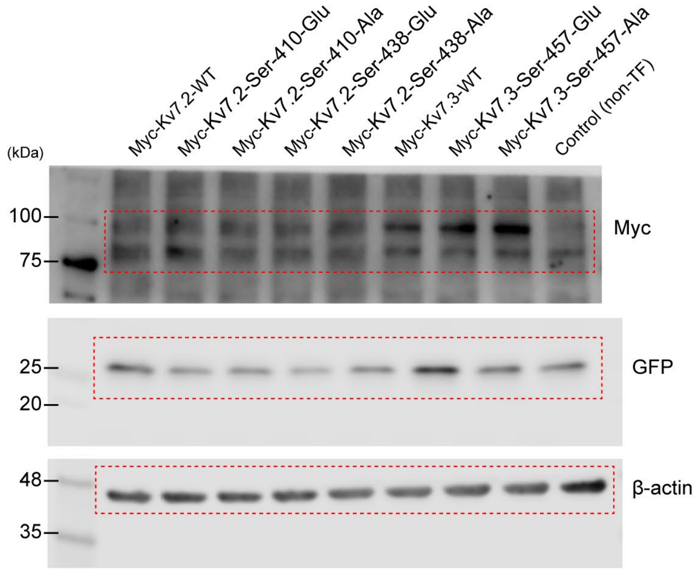

Fig. S7 d uncropped images

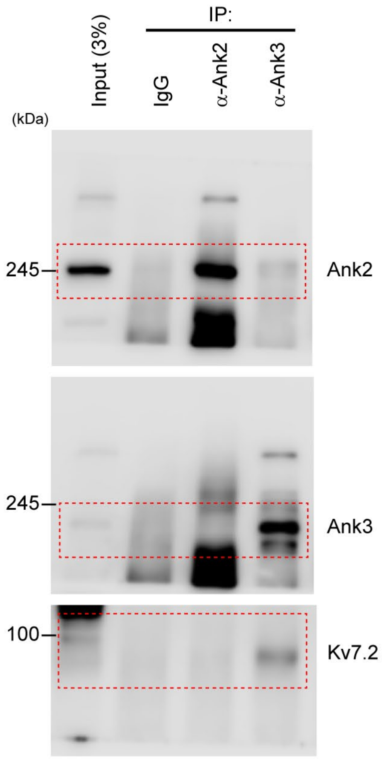

Fig. S7 c uncropped images

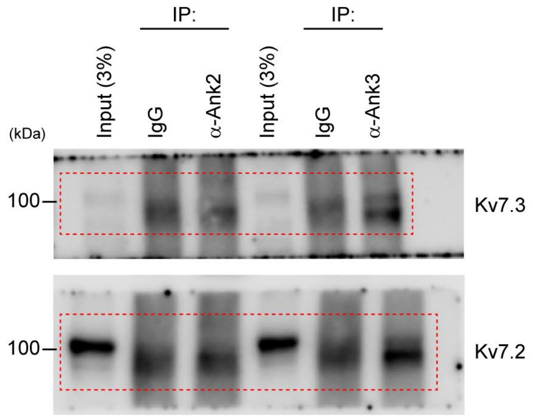

Fig. S13 a uncropped images

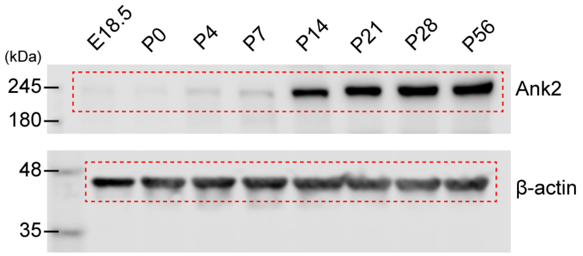

Fig. S13 b uncropped images

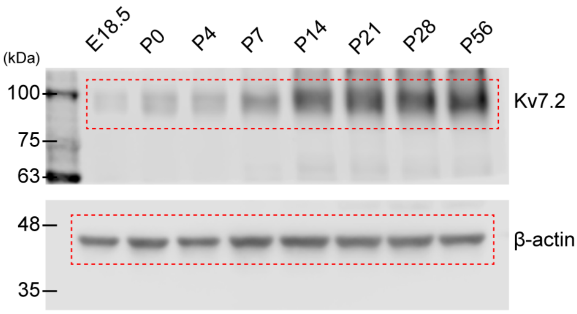

Fig. S13 c uncropped images

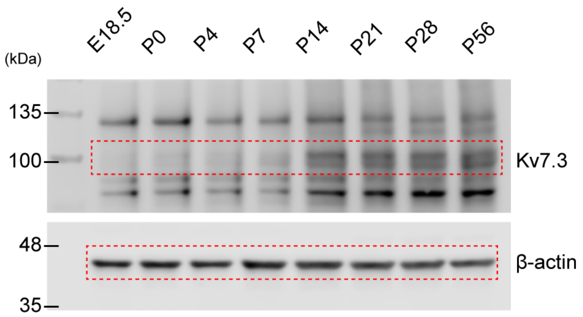

Fig. S13 d uncropped images

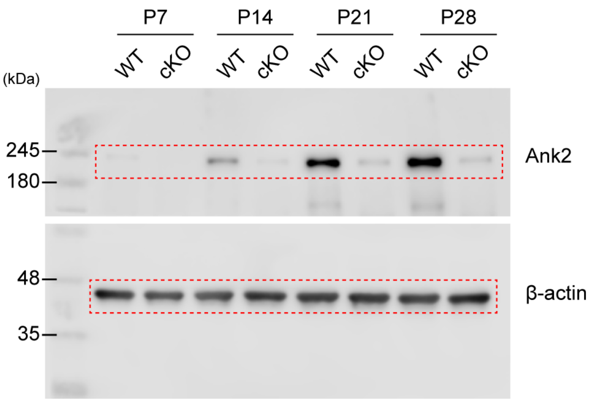

Fig. S13 e uncropped images

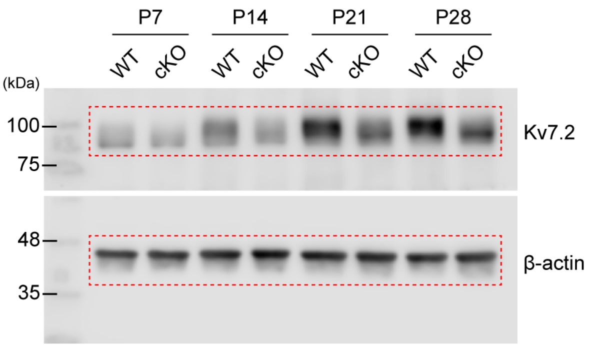

Fig. S13 f uncropped images

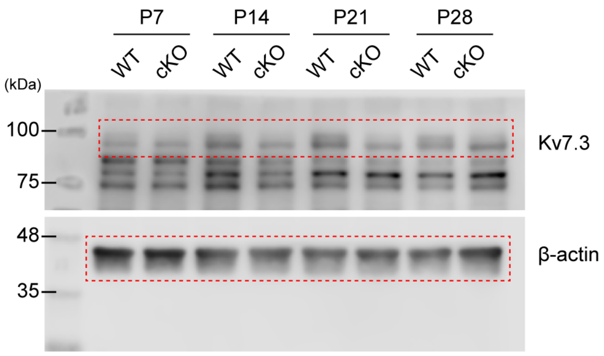

Supplement: Supplementary file 1 — Supplementary Information [file 41467_2023_39203_MOESM1_ESM.pdf]
