# Supplementary material for: Kv7/KCNQ potassium channels in cortical hyperexcitability and juvenile seizure-related death in Ank2-mutant mice
Source: Nat Commun. 2023 Jun 15;14:3547. doi: 10.1038/s41467-023-39203-z (PMC10272139; doi:10.1038/s41467-023-39203-z)
Supplement: Supplementary file 3 — Description of Additional Supplementary Files [file 41467_2023_39203_MOESM3_ESM.pdf]

Description of Additional Supplementary Files

File Name: Supplementary Dataset 1.

Description: Statistical details.

File Name: Supplementary Dataset 2.

Description: List of PTM DEPs from Ank2-cKO mice.

File Name: Supplementary Dataset 3.

Description: List of total DEPs from Ank2-cKO mice.

File Name: Supplementary Dataset 4.

Description: List of synaptosomal DEPs from Ank2-cKO mice.

File Name: Supplementary Dataset 5.

Description: Results of the PSEA.

File Name: Supplementary Dataset 6.

Description: List of gene sets and genes used for ASD-related/risk PSEA.

File Name: Supplementary Dataset 7.

Description: Comparison of mouse phenotypes from the current and previous studies.
