# Supplementary material for: Kv7/KCNQ potassium channels in cortical hyperexcitability and juvenile seizure-related death in Ank2-mutant mice
Source: Nat Commun. 2023 Jun 15;14:3547. doi: 10.1038/s41467-023-39203-z (PMC10272139; doi:10.1038/s41467-023-39203-z)
Supplement: Supplementary file 11 — Reporting Summary [file 41467_2023_39203_MOESM11_ESM.pdf]

nature portfolio

Corresponding author(s): Eunjoon Kim

Last updated by author(s): May 18, 2023

Reporting Summary

Nature Portfolio wishes to improve the reproducibility of the work that we publish. This form provides structure for consistency and transparency in reporting. For further information on Nature Portfolio policies, see our Editorial Policies and the Editorial Policy Checklist.

Statistics

For all statistical analyses, confirm that the following items are present in the figure legend, table legend, main text, or Methods section.

n/a	Confirmed	
☐	☒	The exact sample size (*n*) for each experimental group/condition, given as a discrete number and unit of measurement
☐	☒	A statement on whether measurements were taken from distinct samples or whether the same sample was measured repeatedly
☐	☒	The statistical test(s) used AND whether they are one- or two-sided<br>*Only common tests should be described solely by name; describe more complex techniques in the Methods section.*
☒	☐	A description of all covariates tested
☐	☒	A description of any assumptions or corrections, such as tests of normality and adjustment for multiple comparisons
☐	☒	A full description of the statistical parameters including central tendency (e.g. means) or other basic estimates (e.g. regression coefficient) AND variation (e.g. standard deviation) or associated estimates of uncertainty (e.g. confidence intervals)
☐	☒	For null hypothesis testing, the test statistic (e.g. *F*, *t*, *r*) with confidence intervals, effect sizes, degrees of freedom and *P* value noted<br>*Give P values as exact values whenever suitable.*
☒	☐	For Bayesian analysis, information on the choice of priors and Markov chain Monte Carlo settings
☒	☐	For hierarchical and complex designs, identification of the appropriate level for tests and full reporting of outcomes
☒	☐	Estimates of effect sizes (e.g. Cohen's *d*, Pearson's *r*), indicating how they were calculated

Our web collection on statistics for biologists contains articles on many of the points above.

Software and code

Policy information about availability of computer code

Data collection	Cheetah 5 (Neuralynx)<br>pClamp 10.1 (Molecular Devices)<br>Multiclamp Commander 700B (Molecular Devices)<br>Avisoft SASLab Pro software
Data analysis	Clampfit 10 (Molecular Devices)<br>Matlab 2022a (Mathworks)<br>Ethovision XT 13 (Noldus)<br>Image Studio Lite 3.1 (Li-COR Biosciences)<br>ImageJ (NIH)<br>Imaris 9.7.2<br>Cytoscape (Cytoscape)<br>HALO 2.3 (HALO)<br>Prism7.1.2 (GraphPad)

For manuscripts utilizing custom algorithms or software that are central to the research but not yet described in published literature, software must be made available to editors and reviewers. We strongly encourage code deposition in a community repository (e.g. GitHub). See the Nature Portfolio guidelines for submitting code & software for further information.

Data

Policy information about availability of data

All manuscripts must include a data availability statement. This statement should provide the following information, where applicable:

- Accession codes, unique identifiers, or web links for publicly available datasets
- A description of any restrictions on data availability
- For clinical datasets or third party data, please ensure that the statement adheres to our policy

The data that support the findings of this study and biological materials and custom code used in this manuscript are available from the corresponding authors upon request.

Human research participants

Policy information about studies involving human research participants and Sex and Gender in Research.

Reporting on sex and gender	*Use the terms sex (biological attribute) and gender (shaped by social and cultural circumstances) carefully in order to avoid confusing both terms. Indicate if findings apply to only one sex or gender; describe whether sex and gender were considered in study design whether sex and/or gender was determined based on self-reporting or assigned and methods used. Provide in the source data disaggregated sex and gender data where this information has been collected, and consent has been obtained for sharing of individual-level data; provide overall numbers in this Reporting Summary. Please state if this information has not been collected. Report sex- and gender-based analyses where performed, justify reasons for lack of sex- and gender-based analysis.*
Population characteristics	*Describe the covariate-relevant population characteristics of the human research participants (e.g. age, genotypic information, past and current diagnosis and treatment categories). If you filled out the behavioural & social sciences study design questions and have nothing to add here, write "See above."*
Recruitment	*Describe how participants were recruited. Outline any potential self-selection bias or other biases that may be present and how these are likely to impact results.*
Ethics oversight	*Identify the organization(s) that approved the study protocol.*

Note that full information on the approval of the study protocol must also be provided in the manuscript.

Field-specific reporting

Please select the one below that is the best fit for your research. If you are not sure, read the appropriate sections before making your selection.

[x] Life sciences [ ] Behavioural & social sciences [ ] Ecological, evolutionary & environmental sciences

For a reference copy of the document with all sections, see nature.com/documents/nr-reporting-summary-flat.pdf

Life sciences study design

All studies must disclose on these points even when the disclosure is negative.

Sample size	Sample sizes were determined based on the relevant published literature and/or by the nature of the experimental design. For detail sample size please see figure legends.
Data exclusions	Data with Having normal distribution, we conducted outlier test and p<0.05 data points were excluded.
Replication	All experiments were replicated through multiple cohort/mice analysis, where applicable. We included the results only when the replications lead to the same conclusions.
Randomization	Mice were allocated into specific cohorts at random, except genotype per cage post-weaning was set at a 1:1 ratio for WT vs mutant. Male cohorts were caged separately from females when weaned.
Blinding	All experimenters were blind to the genotype of the mice (sex could not be occluded from the experimenter, due to obviousness of the features). All analyses were performed in a blind manner. Cohorts were grouped at random at time of weaning.

Reporting for specific materials, systems and methods

We require information from authors about some types of materials, experimental systems and methods used in many studies. Here, indicate whether each material, system or method listed is relevant to your study. If you are not sure if a list item applies to your research, read the appropriate section before selecting a response.

Materials & experimental systems

n/a	Involved in the study
☐	☒ Antibodies
☒	☐ Eukaryotic cell lines
☒	☐ Palaeontology and archaeology
☐	☒ Animals and other organisms
☒	☐ Clinical data
☒	☐ Dual use research of concern

Methods

n/a	Involved in the study
☒	☐ ChIP-seq
☒	☐ Flow cytometry
☒	☐ MRI-based neuroimaging

Antibodies

Antibodies used

Mouse anti-Ank2 (StressMarq, SMC-400, 1:500)
Mouse anti-Ank3 (SantaCruzBiotech, sc-12719, 1:1000 for WB, 1:100 for ICC)
Rabbit anti-Ank3 (Synaptic Systems, 386 003, 1:300)
Rabbit anti-Kv1.2 (Millipore, Ab5924, 1:1000)
Mouse anti-Kv2.1 (Neuromab, Ab5924, 1:500)
Rabbit anti-Kv3.1b (Alomone, APC-014, 1:500)
Rabbit anti-Kv7.2 (abcam, ab22897, 1:1000)
Rabbit anti-Kv7.3 (Alomone, APC-051, 1:500)
Rabbit anti-GABA A receptor γ2 (Synaptic Systems, 224 003, 1:1000)
Mouse anti-GABA A receptor β2/3 (Millipore, MAB341, 1:500)
Mouse anti-Gephyrin (Synaptic Systems, 147 111, 1:1000)
Mouse anti-PSD95 (NeuroMab, 75-028, 1:1000)
Rabbit anti-Nav1.2 (Alomone, ASC-002, 1:200)
Rabbit anti-Nav1.6 (Alomone, ASC-009, 1:1000)
Rabbit anti-Caspr (Abcam, ab34151, 1:300)
Anti-mouse GFP (Santa Cruz Biotech, sc-9996, 1:1000)
Anti-mouse Myc (Cell Signaling, 2276, 1:1000)
Chicken anti-mCherry (abcam, ab205402, 1:5000)
Rabbit anti-GAPDH (Cell signaling, 2118, 1:1000)
Mouse anti-GAPDH (Cell signaling, 97166, 1:1000)
Mouse anti-α-tubulin (Sigma, T5168, 1:1000)
Mouse anti-β-actin (Sigma, 5316, 1:1000)
Donkey anti-mouse IgG Alexa Fluor 488 (Thermo, A21202, 1:1000)
Donkey anti-rabbit IgG Alexa Fluor 405 (Thermo, A48258, 1:1000)
Goat anti-chicken IgY Alexa Fluor 594 (Thermo, A11042, 1:1000)
Donkey anti-rabbit IgG Alexa Fluor 594 (Thermo, A21207, 1:1000)
Donkey anti-rabbit IgG Alexa Fluor Plus 647 (Thermo, A32795, 1:300)

Validation

All commercial antibodies have been validated and published, with relevant information existing in the pertaining website.

Animals and other research organisms

Policy information about studies involving animals; ARRIVE guidelines recommended for reporting animal research, and Sex and Gender in Research

Laboratory animals

C56BL/6J strain were used as background of all wildtype/mutant mouse used in the study. All animals were fed ad libitum and housed under 12 h light/dark cycle (light phase from 1 am to 1 pm) under 21 degree celcius and 50-60% humidity.
Our studies involved adult mice (> postnatal day 56) and juvenile (postnatal day 18~28) mice.

Wild animals

The study did not involve wild animals.

Reporting on sex

Data from adult male and female Ank2+/– mice were disaggregated in the data presentation because the results were sexually dimorphic. Data from juvenile male and female Ank2-cKO mice were not disaggregated in the data presentation because males and females showed similar behaviors, but the details on male and female mouse numbers were indicated in the Source Data file. Male Ank2-cKO mice were used for acute slice recording, proteomics analysis, and behavioral rescue experiments because males and females showed similar behaviors.

Field-collected samples

The study did not involve samples collected from the field

Ethics oversight

Mouse maintenance was performed according to the Requirements of Animal Research at KAIST. Experimental procedures for mice were approved by the Committees of Animal Research at KAIST (KA2016-32 and KA2020-91).

Note that full information on the approval of the study protocol must also be provided in the manuscript.
